# Frequency-Guided Expert Modulation for Noisy-Label Facial Expression Recognition

**DOI:** 10.3390/jimaging12080350

**Published:** 2026-08-03

**Authors:** Miaomiao Zhang, Meng Lou, Linwei Chen

**Affiliations:** 1 Zhejiang Polytechnic University of Mechanical and Electrical Engineering, Hangzhou 310053, China; mmzhang1997@yeah.net; 2 School of Computing and Data Science, The University of Hong Kong, Hong Kong SAR, China; 3 Hong Kong Generative AI Research and Development Center, The Hong Kong University of Science and Technology, Hong Kong SAR, China; 4 The Hong Kong University of Science and Technology, Hong Kong SAR, China

**Keywords:** facial expression recognition, noisy labels, frequency-guided modulation

## Abstract

Facial expression recognition in the wild is challenged by both noisy supervision and degraded visual evidence: subtle expression cues must be interpreted under blur, contrast changes, image noise, and annotator disagreement. Existing noisy-label FER methods mainly regulate samples, labels, or attention, while frequency information is rarely used to adapt the semantic representation itself. We propose Frequency-Guided Expert Modulation (FARM-FER), which treats local and global frequency descriptors as a control signal rather than an additional classifier input. A joint Haar-DWT and radial-FFT context guides soft routing among nonlinear experts and channel-wise affine recalibration of the semantic feature, while a learned gate combines the two corrections before a lightweight classifier predicts the expression from the refined representation. Across RAF-DB, FER+, and AffectNet under symmetric label noise, with additional evaluations under class-dependent label noise on RAF-DB and native crowd-label ambiguity on FER+, FARM-FER consistently improves matched baselines. At 30% symmetric noise, FARM-FER reaches 89.18% accuracy on RAF-DB, with a 1.6% performance gain over the matched Swin-Tiny baseline; the gains also hold in a controlled ResNet18 reimplementation and in class-sensitive AffectNet evaluation. Measured cost analyses show only modest parameter and FLOP overhead, supporting a lightweight yet effective design in terms of model size and arithmetic cost for noisy-label FER.

## 1. Introduction

Facial expression recognition (FER) is a central problem in affective computing and image understanding. In-the-wild benchmarks such as RAF-DB, FER+, and AffectNet contain substantial variation in pose, illumination, resolution, occlusion, and annotation source [1,2,3]. Their categories are also intrinsically ambiguous: weak or mixed expressions may receive different labels from different annotators, and a hard training label can conceal this uncertainty [4]. These factors make noisy-label FER more difficult than generic image classification because the supervision error is coupled with subtle local facial evidence and image-quality variation.

Existing noisy-label FER methods mainly intervene at the sample, label, or attention level. SCN and RUL estimate sample reliability, DMUE and UALDL model uncertainty or label distributions, and EAC/AFCN regularize attention and region aggregation [5,6,7,8,9,10]. These methods are effective, but they leave a distinct question open: can observable image-frequency condition control how a semantic expression representation is transformed before classification? This question matters because local expression evidence and global image degradation have different spectral signatures, yet passive feature concatenation does not specify how those signatures should alter the semantic feature.

The discrete wavelet transform (DWT) and fast Fourier transform (FFT) provide complementary views of this problem. Haar DWT preserves spatially localized directional subbands that respond to edges, wrinkles, eyelid contours, and additive high-frequency corruption. A radial FFT descriptor discards angular layout but compactly summarizes the global spectral distribution associated with blur and contrast. Frequency-aware vision methods have used related cues for channel attention, adaptive convolution, feature fusion, and dense prediction [11,12,13,14,15]. However, frequency encoding alone does not establish whether passive fusion, affine conditioning, or conditional transformation is most appropriate for noisy-label FER.

We therefore propose FARM-FER, a frequency-guided expert modulation framework. A normalized joint DWT–FFT context controls two transformations of a Swin-Tiny semantic feature: soft routing over nonlinear experts and continuous affine recalibration. A sample-adaptive gate blends these transformations before residual fusion. The same joint context controls both paths; DWT is not assigned exclusively to routing, nor FFT exclusively to affine recalibration. The raw context is excluded from the final classifier so that the principal comparison concerns active semantic modulation rather than additional classifier input.

The main contributions are:

- We introduce a joint local/global frequency context that controls nonlinear expert routing, affine semantic recalibration, and learned arbitration without exposing raw frequency descriptors to the expression classifier.
- We separate architecture and training effects through a Swin-Tiny 2 × 2 factorial and a ResNet18 controlled reimplementation using the same EAC-style training recipe. Component ablations distinguish descriptor source, modulation path, fusion rule, context size, and expert count with exact parameter accounting.
- We extend evaluation from symmetric corruption to RAF-DB class-dependent noise and FER+ native crowd-label ambiguity, with repeated seeds, macro-F1, balanced accuracy, and per-class analysis.
- We compare FARM-FER with matched SE, CBAM, FiLM, Dynamic/Conditional, and visual-feature MoE adaptations, and report measured parameters, FLOPs, median and P95 latency, peak memory, routing utilization, descriptor correlations, representation separation, and Grad-CAM target-class faithfulness.

Together, the controlled studies establish the central contribution of FARM-FER: in the evaluated still-image FER settings, joint local/global frequency context is more effective when it actively controls semantic transformation than when it is used as passive classifier side information. The accompanying directional-spectrum, routing, and ambiguity analyses also delimit the evidence boundary of this mechanism-level conclusion.

## 2. Related Work

### 2.1. Datasets and Label Ambiguity in In-the-Wild FER

Modern FER evaluation has moved from posed laboratory expressions toward unconstrained images. RAF-DB provides crowd-sourced categorical annotations, FER+ retains annotator vote distributions, and AffectNet combines scale with pronounced class imbalance [1,2,3]. These datasets expose two related but non-identical problems. Synthetic corruption tests robustness under a controlled transition process, whereas vote distributions represent naturally observed disagreement. We therefore evaluate both rather than treating an in-the-wild dataset name alone as evidence of realistic label noise.

### 2.2. Noisy-Label and Uncertainty-Aware FER

General noisy-label learning includes robust losses, sample selection, relabeling, and semi-supervised mixture modeling [16,17]. FER-specific methods exploit task structure: SCN suppresses uncertain samples, DMUE models latent distributions, RUL estimates relative uncertainty, UALDL learns annotation distributions, EAC enforces erasing attention consistency, and AFCN combines certainty analysis, label correction, and attention-fusion consistency [5,6,7,8,9,10]. FARM-FER is complementary to these objectives because it changes the conditional transformation applied to the semantic feature. Our controlled experiments therefore distinguish the FEM architecture from the adopted EAC-style training recipe instead of attributing their combined gain to one factor.

### 2.3. Representation Learning and Attention in FER

Beyond noise handling, FER requires discriminative interactions among subtle facial regions. ResNet, Vision Transformer, DeiT, Swin Transformer, and ConvNeXt provide increasingly strong semantic encoders [18,19,20,21,22], while TransFER adapts relation-aware transformer representations to FER [23]. Because stronger backbones can confound attribution, we use literature-level tables only for field context and rely on matched Swin-Tiny and ResNet18 controls for controlled attribution.

### 2.4. Frequency-Aware Neural Vision

Frequency-aware neural vision spans several mechanisms. FcaNet maps frequency components to channel attention, FADC adapts convolution to frequency characteristics, and FAFF, SFM, and FDAM use spectral cues for feature fusion or dense prediction [11,12,13,14,15]. Recent frequency-aware FER systems use spectral-spatial fusion, wavelet branches, Fourier prompts, or multi-domain frequency enhancement [24,25,26,27]. More generally, SE and CBAM reweight features, FiLM applies conditional affine modulation, Dynamic Convolution selects among kernels, and MoE selects among transformation functions [28,29,30,31,32]. These works motivate the new mechanism taxonomy in Table 1 and the nearest-FER comparison in Table 2.

### 2.5. Position of the Present Work

The primary technical novelty of FARM-FER is an architecture-level coupling in which one joint local/global frequency context simultaneously controls a routed family of nonlinear semantic transformations and continuous channel-wise recalibration, while a learned gate arbitrates their sample-wise contribution and the raw context is withheld from the classifier. This differs from spectral-spatial feature fusion, wavelet-only dual branches, Fourier prompting, frequency attention, conventional affine modulation, and semantic-feature-routed MoE designs in both the source of control and the representation being transformed. The controlled experiments test this contribution through a parameter-matched passive control, parameter-matched single-descriptor controls, diagnostic path ablations with exact parameter counts, matched backbones, alternative noise models, richer metrics, and measured cost.

## 3. Methods

### 3.1. Overview of FARM-FER

Deep FER models trained with hard category labels are prone to overfitting spurious local cues whenever expression evidence is weak or label quality is low. FARM-FER addresses this issue by using compact frequency descriptors as control signals that govern how the semantic backbone feature is transformed, rather than as additional inputs to the classifier.

The architecture comprises three stages. Given an input face image x, the semantic backbone produces a pooled feature $v\in R^{D}$. The primary Swin-Tiny model uses D = 768; the ResNet18 control uses its global-average-pooled D = 512 feature. In parallel, a Haar discrete wavelet transform (DWT) pathway extracts localized frequency structure, and a radial fast Fourier transform (FFT) pathway summarizes the global spectral condition. The DWT encoder produces $d\in R^{96}$, while the FFT encoder produces $z\in R^{48}$. Their concatenation is layer-normalized into $c\in R^{144}$. The 144-dimensional setting was selected from the validation comparison in Section 4.6. The same joint context controls expert routing and affine recalibration; it is not partitioned into a DWT-only routing signal and an FFT-only affine signal. FEM produces a refined semantic feature $\tilde{v}$. The final prediction $\hat{y}\;=\;\mathrm{Cls}\left(\tilde{v}\right)$ is computed without appending c to the classifier. Algorithm 1 specifies the dual-frequency context encoding procedure.

**Figure 1:**
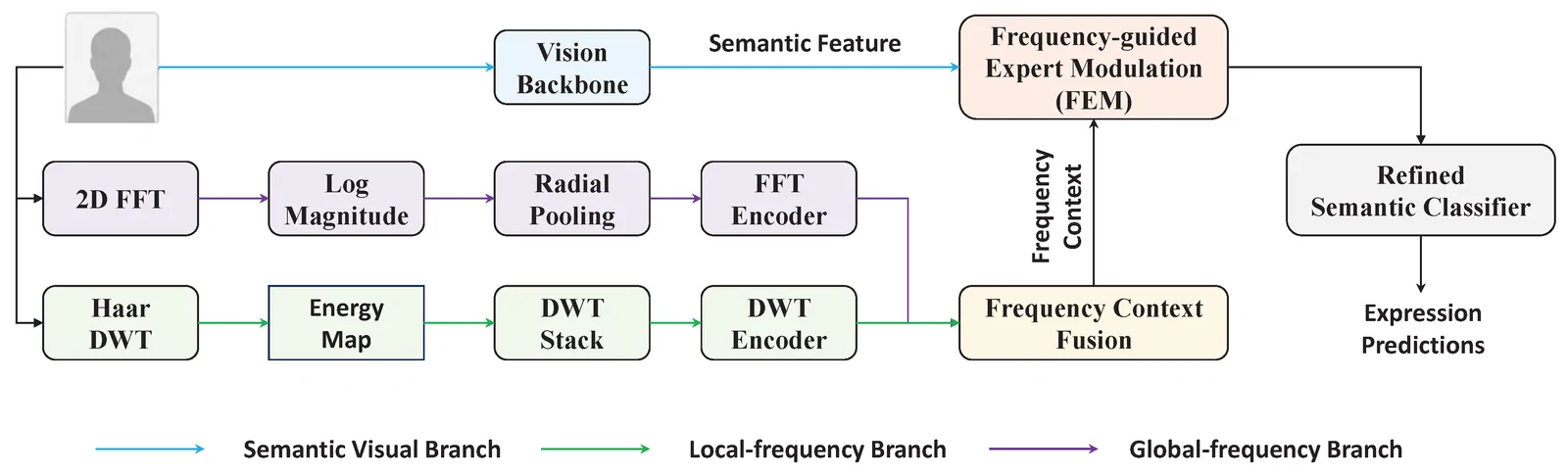
Overall workflow of FARM-FER. The visual branch extracts a semantic feature from the input face, while the local-frequency and global-frequency branches produce DWT and FFT descriptors. The DWT stack is formed from the Haar subbands and the high-frequency energy map, and the FFT branch summarizes the log-magnitude spectrum through radial pooling. Frequency context fusion converts these descriptors into a compact modulation signal that controls the FEM module, and the refined semantic classifier predicts the expression category from the FEM output.

The architecture is summarized in Figure 1.
**Algorithm 1** Procedural specification of dual-frequency context encoding**Require:** 
Mini-batch $x\in R^{B\;\times \;3\;\times \;H\;\times \;W}$, radial-bin count $N_{r}\;=\;16$**Ensure:** 
DWT descriptor $d\in R^{B\;\times \;96}$, FFT descriptor $z\in R^{B\;\times \;48}$, context $c\in R^{B\;\times \;144}$ g←MeanChannel(x) g←EnsureEvenSpatial(g) (LL,LH,HL,HH)←HaarDWT(g) $E\leftarrow \sqrt{LH^{2}\;+\;HL^{2}\;+\;HH^{2}\;+\;\varepsilon }$ S←NormalizePerSample([LL,LH,HL,HH,E]) u←ConvBNReLU(S; 5→16, 3 × 3) u←MaxPool(u) u←ConvBNReLU(u; 16→32, 3 × 3) d←ReLU(Linear(GAP(u); 32→96)) $P\leftarrow \log(1\;+\;|\mathrm{FFTShift}\left(\mathrm{FFT}2_{\mathrm{ortho}}\left(g\right)\right)|)$ **for** i = 1 to $N_{r}$ **do**     $q_{i}\leftarrow {\mathrm{Mean}}_{\rho_{i}\leq \rho <\rho_{i\;+\;1}}\left(P\right)$ **end for** $q\leftarrow [q_{1},\ldots ,q_{N_{r}}]$ $h\leftarrow \mathrm{ReLU}(\mathrm{Linear}\left(q;\;N_{r}\to 48\right))$ z←ReLU(Linear(Dropout(h); 48→48)) c←LN([d; z]) **return** (d,z,c)

### 3.2. Dual-Frequency Context Encoding

Two frequency analyzers operate in parallel on a grayscale projection of the input image, each capturing a distinct aspect of the image’s frequency structure.

**DWT encoder.** The input image x is reduced to a single-channel map *g* by channel averaging. A single-level 2D Haar DWT [33] decomposes *g* into an approximation subband LL and three detail subbands LH, HL, and HH, all at half the spatial resolution of *g*. A high-frequency energy map is computed as

(1) $$E\;=\;\sqrt{LH^{2}\;+\;HL^{2}\;+\;HH^{2}\;+\;\varepsilon }$$

where ε>0 is a numerical stability constant. The five-channel stack [LL,LH,HL,HH,E] is normalized per sample and passed through a lightweight encoder $\Phi_{d}$ consisting of two convolutional blocks with batch normalization and ReLU activation, an interleaved max-pooling step, adaptive average pooling, and a linear projection. This produces the local frequency descriptor $d\in R^{96}$.

**FFT encoder.** The 2D discrete Fourier transform [34] of *g* is computed with orthogonal normalization and frequency-shifted to center the DC component. Log-magnitude coefficients log(|F(g)|+1) are pooled by annular averaging into 16 radial frequency bins, yielding a compact spectral histogram that captures the image’s global frequency bias. This 16-dimensional radial descriptor is mapped through a two-layer MLP to produce the global spectral descriptor $z\in R^{48}$. Radial pooling is compact and rotation-insensitive, but it discards angular spectral layout by construction. Section 4.7 therefore compares it with radial-plus-angular pooling and a lightweight 2D spectrum CNN. For fixed 224 × 224 inputs, normalized annular weights are cached after their first construction; this changes implementation cost but not the descriptor values.

**Context fusion.** The two descriptors are concatenated and layer-normalized:

(2) $$c\;=\;\mathrm{LN}\left(\left[d;\;z\right]\right)\in R^{144}$$

The DWT pathway preserves localized edge and texture responses, whereas the FFT pathway summarizes the holistic spectral distribution. This interpretation is treated as an empirical hypothesis rather than an assumption: Section 4.9 reports correlations with controlled edge, blur, noise, and contrast factors, together with a FER+ vote-entropy probe.

### 3.3. Frequency-Guided Expert Modulation

The FEM module uses the frequency context c to transform the semantic feature v through two complementary pathways: frequency-guided expert routing, which selects among qualitatively different nonlinear transformations, and frequency-conditioned affine recalibration, which applies a smooth global feature adjustment. A sample-adaptive blend gate determines the contribution of each pathway. The blend gate is an arbitration mechanism, not a third transformation pathway.

**Frequency-guided expert routing.** A pool of K = 3 expert networks independently transforms v. The three-expert setting is selected by the validation comparison in Section 4.6. Each expert $E_{k}$ is a two-layer bottleneck network with GELU activation mapping $R^{D}\to R^{192}\to R^{D}$. The frequency context determines a soft routing distribution over experts via a two-layer MLP router $R:R^{144}\to R^{K}$ with GELU activation:

(3) $$\alpha \;=\;\mathrm{softmax}\left(R\left(c\right)\right)\in R^{K},\;\;\;\;r_{\exp}\;=\;\sum_{k\;=\;1}^{K}\alpha_{k}\;E_{k}\left(v\right)$$

Routing from c separates the control signal from the semantic feature being transformed. Softmax provides normalized weights but no theoretical anti-collapse guarantee. We therefore make no such guarantee and instead report empirical utilization, routing entropy, and dominant-expert frequencies in Section 4.9.

**Frequency-conditioned affine recalibration.** In parallel, c produces a channel-wise scale s and shift b for a smooth global adjustment of v. Two-layer MLPs $H_{s},\;H_{b}:R^{144}\to R^{D}$ predict:

(4) $$s\;=\;\tanh\left(H_{s}\left(c\right)\right),\;\;\;b\;=\;H_{b}\left(c\right)$$

(5) $$\Delta v_{\mathrm{aff}}\;=\;0.5\;v⊙s\;+\;b,\;\;\;\;\;v_{\mathrm{aff}}\;=\;v\;+\;\Delta v_{\mathrm{aff}}\;=\;v⊙\left(1\;+\;0.5\;s\right)\;+\;b$$

where ⊙ denotes element-wise multiplication. The factor 0.5 bounds the multiplicative factor to [0.5,1.5], while b supplies the additive correction. We distinguish the affine correction $\Delta v_{\mathrm{aff}}$ from the full affine feature $v_{\mathrm{aff}}$ so that the residual topology is explicit. This pathway provides a continuous, per-channel global adjustment that is complementary to the discrete expert selection.

**Blend-controlled fusion.** A scalar blend gate β interpolates between the routed expert correction and the affine correction on a per-sample basis. A two-layer MLP $H_{\beta }:R^{D\;+\;144}\to R$ with GELU activation, conditioned on both v and c, predicts:

(6) $$\beta \;=\;\sigma (H_{\beta }\left(\left[v;\;c\right]\right))\in R$$

(7) $$\tilde{v}\;=\;\mathrm{LN}\left(v\;+\;\beta \;r_{\exp}\;+\;\left(1-\beta \right)\;\Delta v_{\mathrm{aff}}\right)$$

where σ is the sigmoid function and β is broadcast across feature dimensions. The semantic identity path appears exactly once. At β = 1, the pre-normalization output is $v\;+\;r_{\exp}$; at β = 0, it is $v\;+\;\Delta v_{\mathrm{aff}}\;=\;v_{\mathrm{aff}}$. Intermediate values therefore blend comparable residual corrections before one residual addition and layer normalization. Section 4.5 tests the corresponding expert-only, affine-only, fixed-blend, and learned-blend variants.

**Figure 2:**
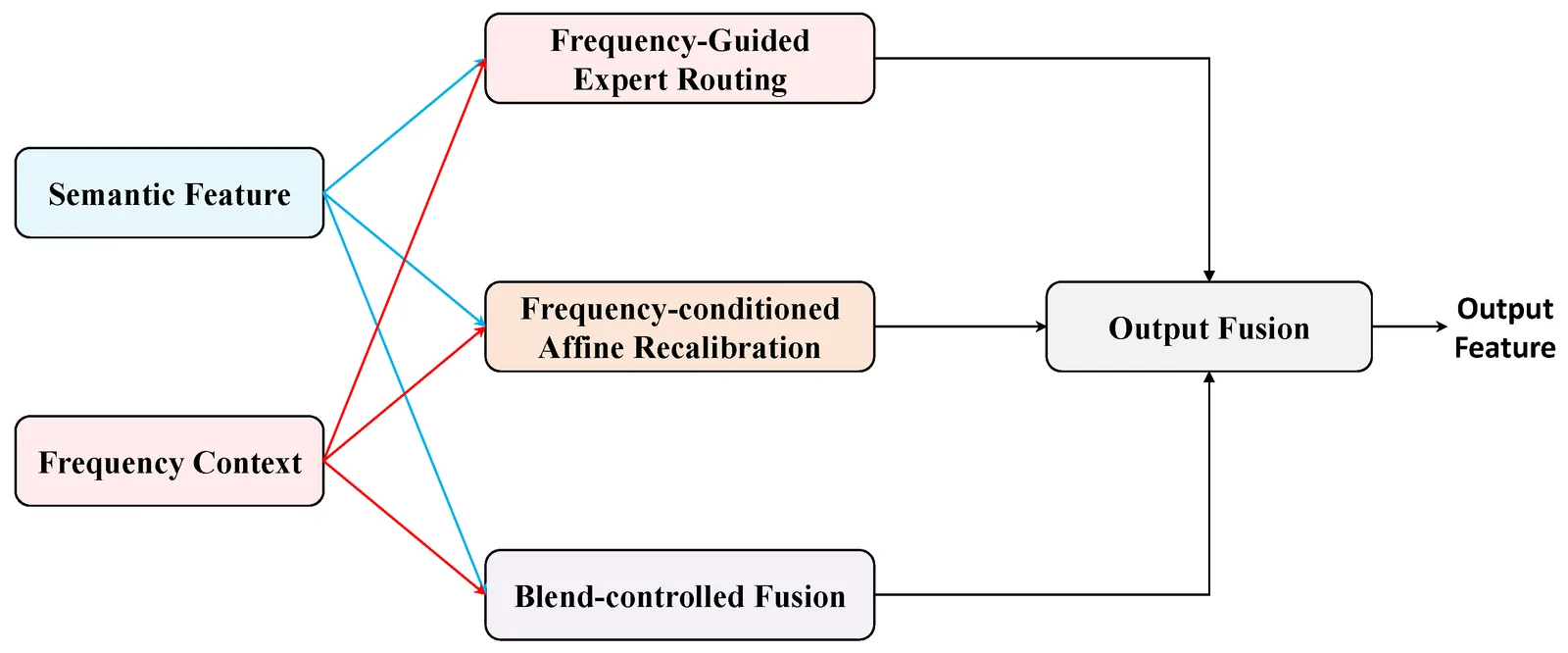
Information flow inside the FEM module. The frequency context controls expert routing and affine recalibration, while the semantic feature and frequency context jointly determine the blend gate before output fusion produces the refined semantic feature. The blend-controlled fusion block arbitrates the two context-conditioned corrections before one residual addition; it is not a third feature-transformation pathway.

Figure 2 is a conceptual information-flow summary rather than a decomposition into three independent transformations. The expert-routing and affine recalibration blocks produce the two context-conditioned corrections. The blend-controlled fusion block does not transform v independently; it computes their sample-wise arbitration before a single residual addition and layer normalization. Thus, the same joint context c conditions both correction branches, while β determines their relative contribution. Equations (3)–(7) and Algorithm 2 give the complete computational sequence.

### 3.4. Refined Semantic Classifier

The refined feature $\tilde{v}$ is passed to a lightweight classifier composed of a dropout layer with rate 0.2 followed by a single linear projection $R^{D}\to R^{7}$, which produces the final expression prediction:

(8) $$\hat{y}\;=\;\mathrm{Cls}\left(\tilde{v}\right)$$

The classifier operates on the refined semantic representation rather than on a direct concatenation of raw frequency descriptors. This removes a direct $c\;\to \;\hat{y}$ branch and keeps the frequency context in its intended controller role inside FEM. Section 4.7 evaluates this architectural choice through linear and deeper heads, a raw-context-only probe, a context-head variant, and classifier-side context interventions. The context-head predictions are sensitive to perturbing the direct context copy, whereas controller-only classification gives the highest observed test mean under the reported protocol.
**Algorithm 2** Procedural specification of frequency-guided expert modulation**Require:** 
Semantic feature $v\in R^{B\;\times \;D}$, context $c\in R^{B\;\times \;144}$, expert set ${\left\{E_{k}\right\}}_{k\;=\;1}^{K}$**Ensure:** 
Refined semantic feature $\tilde{v}$ and classifier input $h_{\mathrm{cls}}$ a←Dropout(GELU(Linear(c; 144→192))) α←softmax(Linear(a; 192→K)) **for** k = 1 to *K* **do**       $p_{k}\leftarrow \mathrm{Dropout}\left(\mathrm{GELU}\left({\mathrm{Linear}}_{k}\left(v;\;D\to 192\right)\right)\right)$       $e_{k}\leftarrow {\mathrm{Linear}}_{k}\left(p_{k};\;192\to D\right)$ **end for** $r_{\exp}\leftarrow \sum_{k\;=\;1}^{K}\alpha_{k}\;e_{k}$ $s\leftarrow \tanh\left({\mathrm{MLP}}^{s}\left(c;\;144\to 192\to D\right)\right),\;b\leftarrow {\mathrm{MLP}}^{b}\left(c;\;144\to 192\to D\right)$ $\Delta v_{\mathrm{aff}}\leftarrow 0.5\;v⊙s\;+\;b$ $\beta \leftarrow \sigma ({\mathrm{MLP}}^{\beta }\left(\left[v;\;c\right];\;\left(D\;+\;144\right)\to 192\to 1\right))$ $o\leftarrow v+\beta r_{\exp}+\left(1-\beta \right)\Delta v_{\mathrm{aff}}$ $\tilde{v}\leftarrow \mathrm{LN}\left(o\right)$ $h_{\mathrm{cls}}\leftarrow \tilde{v}$ **return** $\left(\tilde{v},h_{\mathrm{cls}}\right)$

### 3.5. Experimental Datasets and Noise Protocol

We evaluate RAF-DB, FER+, and AffectNet using their standard seven-class settings [1,2,3]. Before any label corruption, 10% of each official training partition is reserved by class-stratified sampling with fixed split seed 42; the same indices are reused for every model and training seed. For RAF-DB, this gives 11,045 training, 1226 validation, and 3068 official test images. Noise is applied only to the remaining training subset, while validation and test labels remain clean. Symmetric noise replaces a selected training label with a uniformly sampled incorrect class at rates of 10%, 20%, and 30%. To test non-symmetric corruption, RAF-DB class-dependent noise uses a fixed class-dependent confusion map among visually related categories: neutral→sad, sad→neutral, happy→surprise, surprise→fear, fear→surprise, disgust→anger, and anger→disgust. Each selected label follows this predeclared map at the specified corruption rate. Unless otherwise stated, architecture ablations use RAF-DB with 30% symmetric noise.

For FER+ crowd-distribution training, votes for the seven basic emotions are divided by their seven-class sum; samples with zero votes over these classes are excluded. The training objective is soft-target cross-entropy, $-\sum_{j\;=\;1}^{7}p_{j}\log{\hat{p}}_{j}$, without additional label smoothing. Validation checkpoint selection and reported accuracy/macro-F1 use the majority basic-emotion label so that the distribution-trained and hard-label models share the same evaluation target.

### 3.6. Implementation and Training Protocol

FARM-FER is implemented in PyTorch 2.2.2 with ImageNet-1K-pretrained backbones. Inputs are resized to 224 × 224 and normalized with ImageNet mean (0.485,0.456,0.406) and standard deviation (0.229,0.224,0.225). The EAC-style recipe is based on the public EAC implementation and uses the original image together with its horizontally flipped view, random erasing with probability 0.5 and area scale (0.02,0.25), flip-attention consistency, batch size 32, 60 epochs, Adam with $\left(\beta_{1},\beta_{2}\right)\;=\;\left(0.9,0.999\right)$ and $\epsilon \;=\;10^{-8}$, weight decay $10^{-4}$, an exponentially decayed learning rate with factor 0.9 per epoch, and consistency weight 5 [9]. The initial learning rate is $10^{-4}$ for Swin-Tiny and $2\;\times \;10^{-4}$ for ResNet18. Vanilla controls retain the same splits, preprocessing, epochs, optimizer family, and model-selection rule but disable erasing/flip consistency and optimize classification loss only. All Dropout operations shown in Algorithms 1 and 2 use rate 0.2.

The ResNet18 study is a controlled EAC-style reimplementation. Its original fully connected layer is removed, global average pooling produces a 512-D feature, and FEM is inserted immediately before the classifier with D = 512. The 144-D frequency context and 192-unit controller/expert hidden width are unchanged; the scale and shift heads output 512 channels, the blend head receives 512 + 144 inputs, and the classifier is a dropout layer followed by a 512→7 projection. The ResNet18 baseline and ResNet18+FEM models contain 11.180 M and 12.193 M parameters, respectively, and otherwise share the same recipe, splits, seeds, and checkpoint-selection rule.

All matched variants reuse the same corrupted-label file, initialization policy, augmentations, and evaluation code within a seed. The principal RAF-DB 30% comparisons use seeds {7,19,31,43,59}; cross-dataset and alternative-noise results use seeds {7,31,59}. The RAF-DB 30% FARM-FER entry in the field-level table reuses the same five principal runs, whereas its other dataset/noise entries use three runs. Tables report the arithmetic mean and sample standard deviation across seeds unless a caption explicitly identifies pooled per-class or correlation statistics. Paired accuracy intervals are two-sided 95% Student-*t* intervals over seed-wise paired differences. Macro-F1 is the unweighted mean of class-wise F1, and balanced accuracy is the mean class recall. Model selection uses validation accuracy, with macro-F1 as a secondary check on AffectNet; the clean test split is evaluated once per selected run.

Parameter counts are obtained directly from instantiated seven-class models, and FLOPs are computed for a 1 × 3 × 224 × 224 input using the same counting convention for every candidate. End-to-end latency and peak allocated memory are measured under the same FP32 batch-1 setting on an NVIDIA GeForce RTX 4060 Laptop GPU (NVIDIA Corporation, Santa Clara, CA, USA). We report median and P95 latency for the full mechanism sweep and a paired 95% interval for the focused baseline–FARM-FER median-latency comparison.

### 3.7. Compared Methods and Evaluation Metric

Protocol-matched mechanism comparisons adapt SE, CBAM, FiLM, a four-kernel dynamic pointwise transformation, and a three-expert visual-feature MoE to the same final Swin token stage [28,29,30,31,32]. All candidates share the data splits, corrupted-label files, five seeds, optimization schedule, and model-selection rule; Table 3 reports each candidate-specific validation search space, and measured parameter and FLOP counts are reported rather than assumed equal. These are explicitly labeled reimplementations; externally reported costs are not estimated when comparable executable models under the same conditions are unavailable. Baseline, SCN, RUL, EAC, and AFCN provide literature-level context for noisy-label FER [5,7,9,10]. The point estimates used in Table 4 follow the results reported by AFCN [10], while the original methods remain cited in their row labels. These published values are not treated as matched attribution controls because backbones and training pipelines differ. Controlled attribution instead uses the Swin-Tiny and ResNet18 factorials.

## 4. Results

### 4.1. Main Comparison with Noisy-Label FER Methods

Table 4 gives field-level context. The published Baseline, SCN, RUL, EAC, and AFCN point estimates follow the consolidated AFCN comparison and use ResNet-family pipelines, whereas FARM-FER uses Swin-Tiny and repeated runs. Consequently, the table is not used to claim that FEM alone causes every difference. Across the three noise levels, FARM-FER accuracy decreases monotonically as corruption increases and remains above the listed published values. The matched experiments that follow provide the attribution evidence.

### 4.2. Matched Attribution Across Backbones and Training Recipes

Table 5 separates the architecture effect from EAC-style training. Under vanilla training, FARM-FER yields a 1.9% performance gain over Swin-Tiny; under EAC-style training, it yields a 1.6% performance gain over the same backbone. Conversely, EAC-style training improves both tested Swin variants. The non-additive totals indicate a modest interaction, while the positive architecture effect in both recipes shows that the gain is not produced only by EAC-style training. In the ResNet18 controlled reimplementation, adding FEM yields 1.4% and 1.2% performance gains under vanilla and EAC-style training, respectively, providing a second tested architecture context without claiming universal backbone independence.

### 4.3. Accuracy, Macro-F1, and Balanced Accuracy

Table 6 compares the matched backbone, passive frequency concatenation, and FARM-FER at 30% symmetric noise. FARM-FER improves all three metrics on all datasets. The AffectNet macro-F1 gain (2.2%) exceeds its accuracy gain (1.6%), indicating that the improvement is not restricted to majority classes.

The per-class AffectNet F1 values in Table 7 show gains in six of seven classes, with the largest changes on fear and disgust. Anger decreases slightly, which prevents the aggregate result from being interpreted as uniform class-wise dominance.

### 4.4. Class-Dependent Noise and Natural Annotation Ambiguity

Table 8 extends the evaluation beyond symmetric corruption. At 30% class-dependent noise, FARM-FER improves the matched backbone by 1.8% and passive concatenation by 1.3%. FER+ crowd-distribution training yields a 1.0% accuracy gain over the backbone. This setting preserves observed annotator disagreement during optimization, while validation and the reported accuracy/macro-F1 use the common majority-label endpoint for a controlled comparison with hard-label training. These settings are complementary: the class-dependent protocol remains synthetic, whereas FER+ supplies naturally observed ambiguity without being presented as a complete distribution-calibration evaluation.

### 4.5. Component Decomposition

Table 9 isolates descriptor source, transformation path, fusion rule, and model capacity while reporting the exact parameter count of every row. The original passive concatenation gives a 0.4% performance gain over the backbone. The parameter-matched passive adapter concatenates the 768-D visual feature and 144-D context, applies a 912→859→768 GELU MLP with 0.2 dropout, and then uses the same linear classifier. It matches FARM-FER at 28.982 M parameters to the reported precision and reaches 88.24 ± 0.21%, but remains 0.9% below active modulation; the paired five-seed difference is +0.9% with a 95% confidence interval of [0.8%,1.1%]. DWT-only and FFT-only controls are each projected to the same 144-dimensional control space and parameter-matched to FARM-FER. Both are useful, while the joint context exceeds the better single-frequency variant by 0.8%. The expert-only, affine-only, and fixed-blend rows are diagnostic topology ablations with naturally different parameter counts; their exact capacities are therefore shown rather than treated as matched effect sizes. Their observed ordering supports complementary pathways, and the learned model is 0.4% above the fixed blend.

### 4.6. Context Dimension and Expert Count

The capacity study in Table 10 reports both validation and test accuracy. The 144-dimensional context and three-expert bank are selected by mean validation accuracy before test evaluation. Increasing context dimension or expert count beyond these values does not monotonically improve either split.

### 4.7. FFT Representation, Path Assignment, and Classifier Design

Table 11 addresses five design questions. First, adding angular sectors gives a 0.1% mean increase over radial pooling, with a paired 95% confidence interval of [−0.1%,0.2%]; the 2D spectrum CNN differs by −0.1% with interval [−0.3%,0.1%]. These intervals do not resolve an advantage over radial pooling, which is retained as the compact option while its information loss is acknowledged. Second, split DWT/FFT assignments are weaker than allowing the joint context to control both paths. Third, deeper classifier heads do not improve test accuracy and show larger absolute validation–test gaps. Fourth, raw context is label-informative on its own, and the classifier-side intervention shows that context-head predictions are sensitive to the added direct copy; the controller-only model has the highest observed test mean. Finally, the early-fusion control concatenates RGB, resolution-aligned Haar subbands, and the 2D log-spectrum map before one shared image encoder and uses the same training protocol. Because its topology is not parameter-matched, it is interpreted only as a branch-layout control; its lower accuracy supports branch separation as an empirical inductive bias rather than a theoretical necessity.

Context intervention isolates the role of the direct classifier branch (Table 12). Perturbing the FEM context reduces the controller-only model from 89.18% to 87.83% when shuffled and 87.69% when zeroed. To isolate the added classifier path, the context-head model is evaluated with the correct context retained inside FEM while only the classifier copy is shuffled or zeroed; accuracy decreases from 88.75% to 87.92% and 87.78%, respectively. Thus, predictions in the context-head model are sensitive to the direct context copy under this controlled intervention. The same context-head model is 0.4% below the controller-only model, and perturbing both copies reduces accuracy further to 86.96% and 86.81%. These controls support retaining the frequency context as a semantic-modulation signal rather than exposing it directly to the classifier.

### 4.8. Matched Modulation Baselines and Measured Cost

Table 13 compares mechanisms at the same final Swin token stage using the configurations in Table 3. SE and CBAM perform channel/spatial reweighting, FiLM uses the same frequency context for affine conditioning, Dynamic uses a four-kernel pointwise transformation, and visual MoE routes from the semantic feature. FARM-FER achieves the highest accuracy under the shared protocol, and the table quantifies its parameter, FLOP, latency, and memory profile without implying equal parameter counts.

All costs are measured from executable seven-class models. FARM-FER adds 1.457 M parameters (5.29%) and increases FLOPs from 4.5084 to 4.5349 GFLOPs (0.59%). In the full sweep, median latency increases from 14.94 to 19.85 ms (32.9%), P95 latency from 29.14 to 60.51 ms, and peak allocated memory from 134.59 to 143.56 MiB. The focused two-model run gives medians of 13.728 and 17.473 ms, a 27.3% increase with a paired-bootstrap 95% confidence interval of 20.4–40.8%. Thus, the matched 1.6% performance gain is obtained with modest parameter and FLOP increases and a measured central- and tail-latency tradeoff. External SCN/RUL/EAC/AFCN latency is not compared because equivalent executable models under the same conditions are unavailable.

### 4.9. Mechanism and Interpretability Evidence

**Figure 3:**
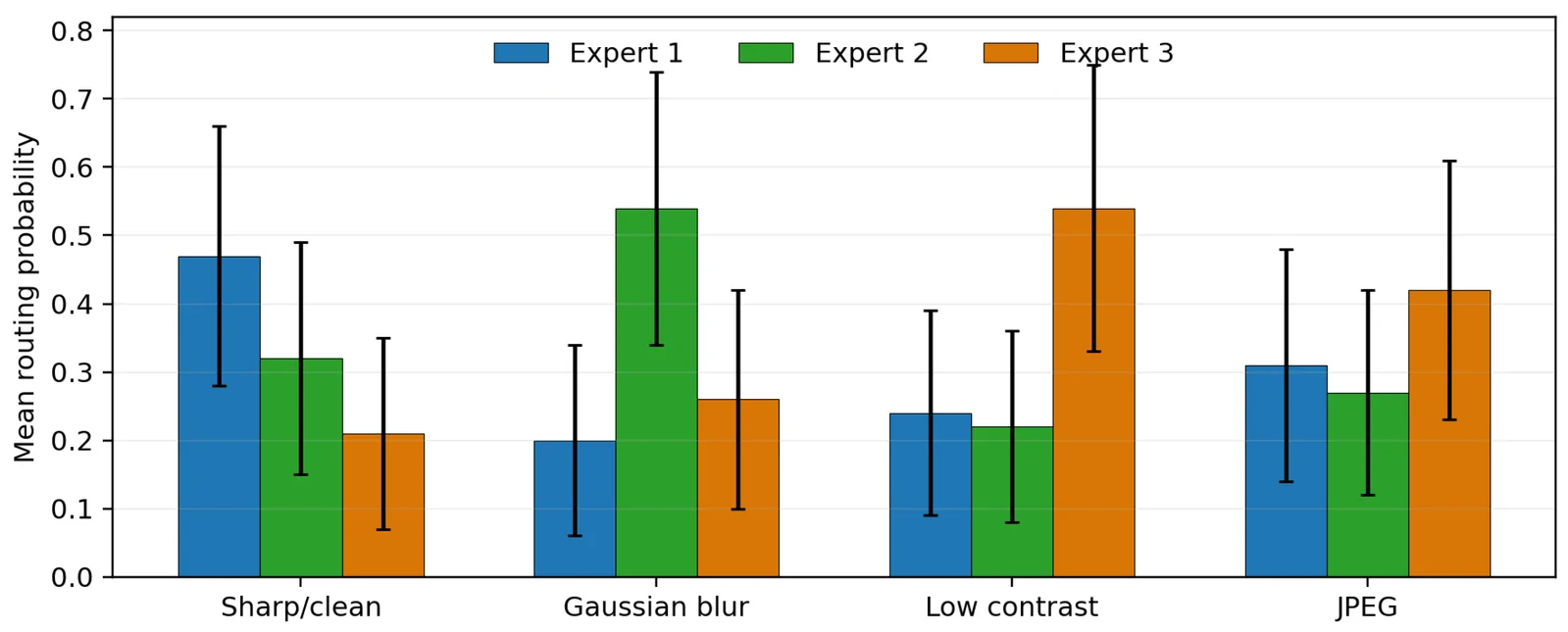
Illustrative expert-routing profiles from the seed-31 checkpoint under controlled image conditions. Error bars denote standard deviation across samples within each condition; the separate across-seed collapse audit is reported in the text.

**Routing-profile illustration and collapse audit.** Table 14 and Figure 3 use a fixed class-stratified set of 1000 RAF-DB test images and the seed-31 checkpoint. Each image is evaluated unmodified, with Gaussian blur (σ = 2), with contrast multiplied by 0.5, and after JPEG compression at quality 30; the reported standard deviations are across images within each condition. At this checkpoint, sharp images shift mass toward Expert 1, blurred images toward Expert 2, and low-contrast images toward Expert 3. These are illustrative group-level tendencies rather than across-seed estimates or fixed semantic labels for the experts. A separate five-checkpoint audit computes one summary per training seed: dominant-expert frequencies are 38.6 ± 2.8%, 34.2 ± 2.4%, and 27.2 ± 2.1%; mean per-sample routing entropy is 0.93 ± 0.03 nats compared with ln(3) = 1.10; and 3.8 ± 0.7% of samples have maximum routing probability above 0.90. Mean pairwise cosine similarities between expert residual outputs are 0.42 ± 0.04, 0.37 ± 0.05, and 0.45 ± 0.04. Together, the across-seed diagnostics show non-collapsed routing and non-identical expert outputs in the evaluated runs, while the fixed-checkpoint profile provides a concrete illustration of condition-dependent routing behavior.

**Descriptor sensitivity.** The protocol uses 1000 RAF-DB test images. Gaussian blur uses σ∈{0,0.5,1.0,1.5,2.0} pixels; zero-mean additive Gaussian noise uses standard deviation {0,0.02,0.04,0.06,0.08} on images scaled to [0,1]; and contrast uses multiplicative factors {1.0,0.8,0.6,0.4,0.2}. Local edge density is the mean Sobel magnitude inside the aligned face crop. The scalar DWT statistic is the spatial mean of the pre-normalization high-frequency energy map in Equation (1); the scalar FFT statistic is the mean log magnitude in the outer eight of the 16 radial bins. Spearman intervals use 2000 image-cluster bootstrap samples. Learned-descriptor probes are ridge regressions fitted on frozen 96-D DWT or 48-D FFT descriptors with image-disjoint five-fold evaluation. Table 15 shows that DWT statistics and learned features have a stronger association with local edge density and additive-noise severity, whereas FFT statistics and features more strongly encode global blur and contrast reduction. For FER+, normalized seven-class Shannon vote entropy defines the ambiguity target, with the top quartile labeled high entropy; AUROC is evaluated in image-disjoint five folds over all eligible test images. The joint descriptor obtains 0.69 ± 0.016, compared with 0.63 ± 0.018 and 0.65 ± 0.021 for DWT and FFT alone. This moderate value shows that frequency context only partially reflects annotation uncertainty.

**Representation structure, frequency probes, and target-class explanation faithfulness.** Representation metrics use the L2-normalized features of all 3068 RAF-DB test images. Silhouette score uses the seven ground-truth classes; intra-class distance is the mean distance to the corresponding class centroid; inter-class distance is the mean pairwise class-centroid distance; and Fisher separation is the inter-class to intra-class ratio. Distances and Fisher values are normalized to the matched backbone within each seed before aggregation. Frozen ridge probes recover blur, contrast, DWT-energy, and FFT-ratio targets from v and $\tilde{v}$ using image-disjoint five-fold evaluation. Grad-CAM [35] metrics use the same fixed class-stratified 1000-image set for each seed, the ground-truth class score, 100 rank-ordered reveal/removal steps, and a Gaussian-blurred reference image. Perturbation stability is the correlation between normalized maps before and after a two-pixel translation plus a 5% brightness change, after inverse alignment. Table 16 reports mean ± sample standard deviation across the five seed checkpoints for every row, and Table 17 reports the corresponding frozen-probe results.

**Figure 4:**
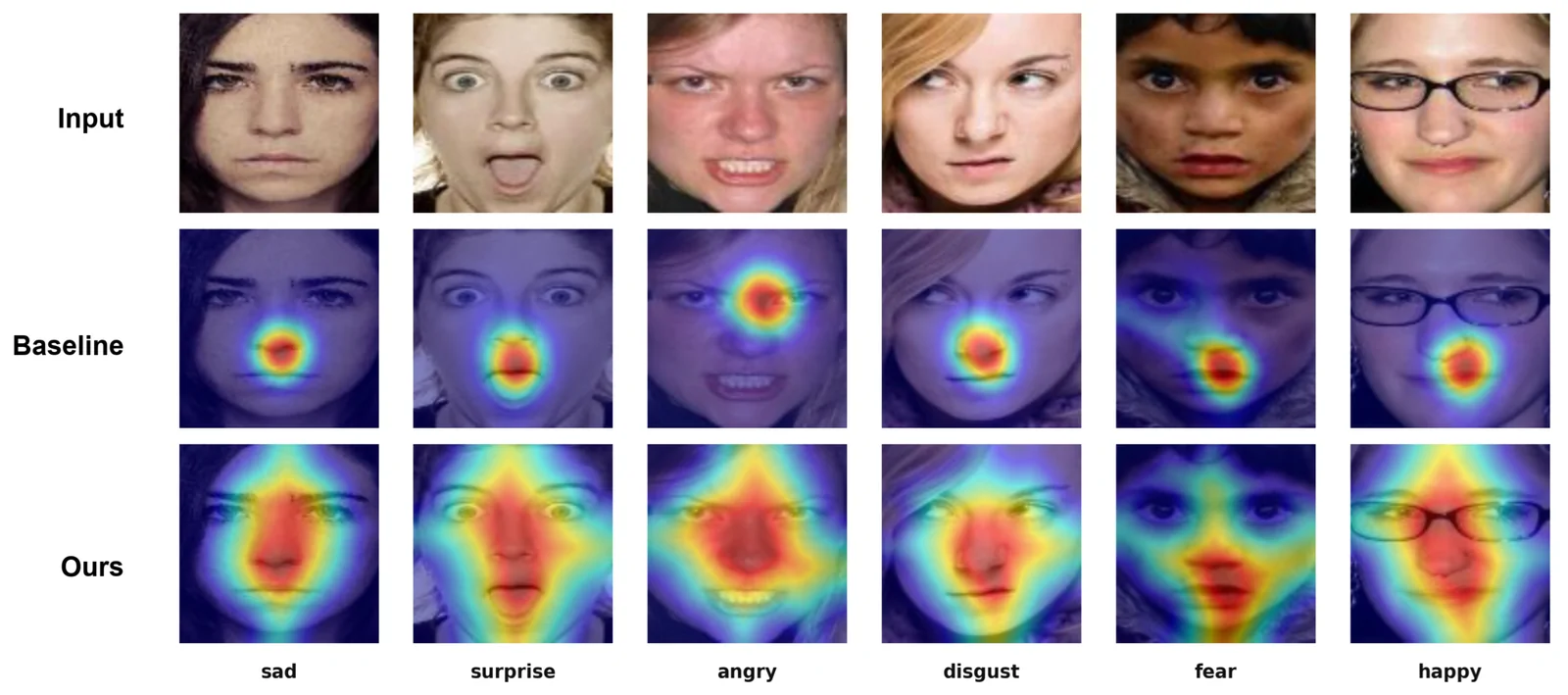
Qualitative Grad-CAM comparison on RAF-DB. The first row shows inputs, the second the matched Swin-Tiny baseline, and the third FARM-FER. The figure is interpreted together with the quantitative ground-truth-class faithfulness metrics in Table 16. Warmer colours indicate a larger contribution to the ground-truth class score and cooler colours a smaller contribution.

Figure 4 provides representative qualitative examples. Several FARM-FER examples show broader central-face coverage than the matched baseline; the quantitative claim is based on the insertion, deletion, and stability measurements in Table 16, not on the examples alone.

## 5. Discussion

### 5.1. Why Active Frequency-Guided Modulation Helps

The main empirical distinction is between adding frequency dimensions and using frequency context to control transformation. Passive concatenation yields a 0.4% performance gain over the matched backbone, whereas FARM-FER yields a 1.6% performance gain and exceeds passive concatenation by 1.2%. Increasing passive-fusion capacity to match FARM-FER raises the passive result to 88.24 ± 0.21%, but active modulation remains 0.9% higher with a paired 95% confidence interval of [0.8%,1.1%]. The same ordering appears under class-dependent noise. Equal-dimensional, parameter-matched DWT-only and FFT-only controls show that the joint-context gain is not explained by input width. The path-only and fixed-blend rows then provide topology diagnostics with their exact, non-identical parameter counts reported explicitly.

The descriptor analysis provides qualified support for the local/global decomposition. Both handcrafted statistics and learned-descriptor probes show stronger DWT sensitivity to local edge density and additive high-frequency noise, and stronger FFT sensitivity to global blur and contrast reduction. Frozen probes from the refined semantic feature recover these frequency condition targets more accurately than probes from the backbone feature, while the moderate FER+ vote-entropy AUROC shows that frequency context is not a direct estimator of label correctness. The defensible interpretation is that the descriptors characterize image conditions that can influence the reliability of semantic evidence.

### 5.2. Relation to Prior Noisy-Label FER Mechanisms

FARM-FER intervenes at the representation-transformation level. SCN/RUL regulate sample reliability, EAC regularizes attention consistency, and AFCN combines certainty and fusion consistency. The 2 × 2 factorial shows positive FEM effects under both tested Swin-Tiny training recipes, with a modest interaction rather than simple additivity. The ResNet18 controlled reimplementation supplies a second architecture context under the same EAC-style recipe without implying universal backbone independence.

The nearest-work and protocol-matched mechanism comparisons clarify and substantiate the technical novelty. Recent frequency-aware FER systems fuse spectral-spatial branches, wavelet features, Fourier prompts, or multi-domain frequency features, while SE/CBAM reweight existing evidence, FiLM provides continuous conditioning, and visual MoE selects transformations from semantic features. The primary technical novelty of FARM-FER is an architecture-level coupling in which a joint DWT–FFT condition controls both nonlinear expert selection and channel-wise affine recalibration, with sample-wise arbitration between the two transformations and no direct raw-context path to the classifier. The controlled comparisons show that this integration is more effective than passive frequency fusion, parameter-matched passive adaptation, single-path modulation, and protocol-matched implementations of the nearest mechanism families in the evaluated noisy-label FER setting.

### 5.3. Limitations and Practical Scope

Several limitations define the present scope. First, class-dependent corruption remains synthetic, and FER+ vote distributions represent one form of natural ambiguity rather than all real annotation processes. Second, radial FFT pooling intentionally removes angular layout. The directional alternative has no resolved advantage here, but tasks that depend on orientation-specific spectral structure may require a different representation. Third, soft routing has no theoretical anti-collapse guarantee; the utilization, entropy, and expert-output analyses establish empirical non-collapse for the evaluated runs. Fourth, the classifier-side interventions establish direct-branch reliance under the evaluated architecture and test protocol; the controller-only design is therefore selected on the corresponding controlled accuracy evidence.

The measured cost defines the deployment profile. FARM-FER adds 5.29% parameters, 0.59% FLOPs, and 8.97 MiB peak allocated memory. Median FP32 batch-1 latency increases by 32.9% in the full mechanism sweep and by 27.3% in the focused two-model protocol; the latter has a bootstrap 95% interval of 20.4–40.8%. Full-sweep P95 latency increases from 29.14 to 60.51 ms. Deployment optimization may reduce this overhead.

Finally, the study concerns still-image categorical FER. It does not evaluate video dynamics, multimodal affect, domain shift across acquisition devices, or clinical cohorts. These are appropriate directions for extending the modulation principle, but the current evidence should not be generalized to them without additional experiments.

## 6. Conclusions

This paper presented FARM-FER, in which a joint DWT–FFT context controls soft expert routing, affine recalibration, and learned blending of two residual corrections to a semantic FER representation. At 30% symmetric noise, the model reaches 89.18 ± 0.16% on RAF-DB, 88.76 ± 0.20% on FER+, and 59.74 ± 0.26% on AffectNet. Matched experiments show a 1.6% performance gain over Swin-Tiny/EAC-style, a 0.9% advantage over a parameter-matched passive adapter, and a 1.2% performance gain in a ResNet18 controlled reimplementation using the same EAC-style recipe. The active design retains gains under RAF-DB class-dependent noise and FER+ crowd-label distributions.

Component decomposition with matched passive and single-descriptor controls, exact parameter accounting, routing analysis, descriptor probes, representation metrics, and explanation measurements supports the intended mechanism without implying theoretical guarantees. FARM-FER offers a measurable accuracy benefit with a quantified efficiency profile: 5.29% more parameters, 0.59% more FLOPs, and a 27.3–32.9% median FP32 batch-1 latency increase across the two reported protocols. Within these boundaries, the results support joint frequency context as a useful control signal for semantic transformation in noisy-label still-image FER.

## Figures and Tables

**Table 1:** Mechanism-level position of FARM-FER. The table identifies the signal that controls adaptation and the operation applied to the semantic representation.

Family	Control Signal	Primary Operation	Difference from FARM-FER
SE/CBAM	Visual feature statistics	Channel/spatial reweighting	No explicit DWT–FFT condition or transformation bank
FiLM	External or learned condition	Continuous scale and shift	No routed family of nonlinear experts
Dynamic convolution	Visual condition	Kernel mixture	Changes convolution kernels rather than a pooled FER representation
Visual-feature MoE	Semantic visual feature	Expert selection	Routing is derived from the representation being transformed
Passive frequency fusion	DWT/FFT descriptor	Classifier concatenation	Frequency cues bypass active semantic transformation
FARM-FER	Joint DWT–FFT context	Expert routing + affine recalibration + learned blend	Frequency condition controls two complementary transformations and is excluded from the classifier input

**Table 2:** Nearest frequency-aware FER methods and the specific technical boundary of FARM-FER. “Noisy labels” denotes explicit training or evaluation under corrupted or ambiguous supervision, rather than only in-the-wild acquisition.

Method	FER Setting	Published Frequency Mechanism	Difference from FARM-FER
SPAYOLO [24]	Real-time FER	FFT frequency-enhancement path with learnable spectral reweighting, channel attention, and gated spatial–frequency fusion	Frequency features are fused within a YOLOv8 representation; no joint DWT–FFT control of routed experts and affine recalibration, and no noisy-label study
FPNet [25]	In-the-wild FER	DWT branch with cross-attention fusion to a spatial branch	Wavelet-only dual-branch fusion; no joint DWT–FFT controller or nonlinear expert bank
NR-GFER [26]	Cross-domain FER	Fourier prompt with prompt adaptation and cross-stage fusion	Frequency prompting guides reconstruction and transfer; no dual-path expert/affine modulation under label noise
MDFEFT [27]	In-the-wild FER	Multi-domain frequency features with adaptive multi-branch enhancement	Frequency-enhanced representation fusion; no routed nonlinear experts or raw-context exclusion test
FARM-FER	Noisy-label FER	Joint Haar-DWT and radial FFT context controlling expert routing, affine recalibration, and a learned blend	Frequency context controls two semantic transformations and is withheld from the classifier input

**Table 3:** Exact configurations for the protocol-matched mechanism comparison. Each candidate uses the same insertion stage, five seeds, training protocol, and model-selection rule; the candidate-specific search space and validation-selected setting are listed explicitly.

Mechanism	Selected Implementation	Validation Search
SE	Final-token channel gate, reduction 16	Reduction {8,16}
CBAM	Shared channel MLP, reduction 16; 7 × 7 spatial gate on the 7 × 7 token map	Reduction {8,16}; spatial kernel {3,7}
Frequency-conditioned FiLM	Joint 144-D context; hidden width 192; bounded scale plus shift	Hidden width {96,192,384}
Dynamic/Conditional	Four learned 1 × 1 token transforms; visual router width 192	Kernels {2,4}; router width {96,192}
Visual-feature MoE	Three residual MLP experts; visual router; expert width 192	Experts {2,3,4}; width {96,192}
FARM-FER	Three residual MLP experts; context router; affine heads; learned blend; width 192	Experts and context dimensions selected in Section 4.6

**Table 4:** Field-level comparison under symmetric label noise. Published point estimates for Baseline, SCN, RUL, EAC, and AFCN follow the results reported by AFCN [10], with original-method citations retained in the row labels. FARM-FER uses three seeds except for RAF-DB at 30%, which reuses the five principal matched runs. Different backbones and training pipelines preclude causal attribution from this table alone.

Method	Noise	RAF-DB	FER+	AffectNet
Baseline	10%	81.01	83.29	57.24
SCN [5]	10%	82.15	84.99	58.60
RUL [7]	10%	86.17	86.93	60.54
EAC [9]	10%	88.02	87.03	61.11
AFCN [10]	10%	82.15	84.99	58.60
FARM-FER	10%	90.27 ± 0.14	90.14 ± 0.13	63.33 ± 0.19
Baseline	20%	77.98	82.34	55.89
SCN [5]	20%	79.79	83.35	57.51
RUL [7]	20%	84.32	85.05	59.01
EAC [9]	20%	86.05	86.07	60.29
AFCN [10]	20%	87.78	88.50	59.58
FARM-FER	20%	89.77 ± 0.18	89.82 ± 0.17	62.49 ± 0.22
Baseline	30%	75.50	79.77	52.16
SCN [5]	30%	77.45	82.20	54.60
RUL [7]	30%	82.06	83.90	56.93
EAC [9]	30%	84.42	85.44	58.31
AFCN [10]	30%	87.45	87.83	58.71
FARM-FER	30%	89.18 ± 0.16	88.76 ± 0.20	59.74 ± 0.26

Bold indicates the best result in each comparison; the same convention applies to all subsequent tables.

**Table 5:** Architecture × training-recipe factorial on RAF-DB with 30% symmetric noise. All entries are mean ± standard deviation over five seeds.

Architecture	Training Recipe	Accuracy (%)
Swin-Tiny backbone	Vanilla	85.92 ± 0.27
FARM-FER (Swin-Tiny)	Vanilla	87.81 ± 0.29
Swin-Tiny backbone	EAC-style	87.58 ± 0.22
FARM-FER (Swin-Tiny)	EAC-style	89.18 ± 0.16
ResNet18	Vanilla	84.73 ± 0.31
ResNet18 + FEM	Vanilla	86.11 ± 0.34
ResNet18	EAC-style	86.84 ± 0.28
ResNet18 + FEM	EAC-style	88.05 ± 0.23

**Table 6:** Matched evaluation at 30% symmetric noise. RAF-DB results use five seeds; FER+ and AffectNet use three. BA denotes balanced accuracy.

Dataset	Variant	Accuracy	Macro-F1	BA
RAF-DB	Backbone	87.58 ± 0.22	86.91 ± 0.25	87.04 ± 0.24
RAF-DB	Passive concat	88.02 ± 0.15	87.42 ± 0.29	87.51 ± 0.18
RAF-DB	FARM-FER	89.18 ± 0.16	88.67 ± 0.21	88.73 ± 0.23
FER+	Backbone	87.29 ± 0.24	86.75 ± 0.27	86.88 ± 0.26
FER+	Passive concat	87.70 ± 0.19	87.16 ± 0.31	87.25 ± 0.21
FER+	FARM-FER	88.76 ± 0.20	88.24 ± 0.22	88.31 ± 0.25
AffectNet	Backbone	58.10 ± 0.31	52.84 ± 0.36	53.17 ± 0.34
AffectNet	Passive concat	58.62 ± 0.24	53.56 ± 0.29	53.91 ± 0.38
AffectNet	FARM-FER	59.74 ± 0.26	55.08 ± 0.34	55.41 ± 0.30

**Table 7:** AffectNet per-class F1 (%) at 30% symmetric noise. Each entry is the mean class-wise F1 over the three dataset seeds, and the class-wise mean equals the macro-F1 in Table 6.

Variant	Neutral	Happy	Sad	Surprise	Fear	Disgust	Anger	Mean
Backbone	64.32	70.48	50.91	56.83	39.72	42.86	44.76	52.84
Passive concat	64.88	70.83	51.74	57.29	40.91	44.13	**45.14**	53.56
FARM-FER	**66.11**	**71.64**	**53.82**	**59.21**	**43.67**	**47.02**	44.09	**55.08**

**Table 8:** Evaluation beyond symmetric noise. Values are mean ± standard deviation over three seeds.

Setting	Variant	Noise/Target	Accuracy	Macro-F1
RAF-DB class-dependent	Backbone	20%	86.74 ± 0.27	86.09 ± 0.30
RAF-DB class-dependent	Passive concat	20%	87.21 ± 0.18	86.62 ± 0.34
RAF-DB class-dependent	FARM-FER	20%	88.42 ± 0.23	87.83 ± 0.25
RAF-DB class-dependent	Backbone	30%	85.39 ± 0.33	84.71 ± 0.36
RAF-DB class-dependent	Passive concat	30%	85.97 ± 0.22	85.32 ± 0.31
RAF-DB class-dependent	FARM-FER	30%	87.23 ± 0.27	86.61 ± 0.35
FER+ native ambiguity	Backbone	Majority labels	88.59 ± 0.18	87.03 ± 0.27
FER+ native ambiguity	FARM-FER	Majority labels	89.71 ± 0.22	88.21 ± 0.24
FER+ native ambiguity	Backbone	Crowd distribution	89.01 ± 0.23	87.48 ± 0.20
FER+ native ambiguity	FARM-FER	Crowd distribution	89.96 ± 0.19	88.68 ± 0.26

**Table 9:** Component decomposition with explicit parameter and FLOP accounting on RAF-DB with 30% symmetric noise. Accuracy is mean ± standard deviation over five seeds. Parameter matching applies only to rows labeled parameter-matched; exact costs are shown for all variants. Latency is full-sweep median FP32 batch-1 latency.

Context/Mechanism	Accuracy (%)	Params (M)	GFLOPs	Median ms
Backbone	87.58 ± 0.22	27.525	4.5084	14.94
Passive DWT + FFT concatenation	88.02 ± 0.15	27.538	4.5334	18.61
Parameter-matched passive adapter	88.24 ± 0.21	28.982	4.5349	17.42
DWT-only, 144-D parameter-matched FEM	88.34 ± 0.19	28.982	4.5341	19.47
FFT-only, 144-D parameter-matched FEM	88.21 ± 0.23	28.982	4.5107	19.20
Joint context, expert path only	88.52 ± 0.24	28.454	4.5343	18.85
Joint context, affine path only	88.43 ± 0.17	27.890	4.5338	17.93
Joint context, expert+affine, fixed β = 0.5	88.82 ± 0.22	28.807	4.5347	18.86
Joint context, full learned FEM	89.18 ± 0.16	28.982	4.5349	19.85

**Table 10:** Context-dimension and expert-count sensitivity on RAF-DB with 30% symmetric noise. Values are mean ± standard deviation over five seeds.

Study	Configuration	Validation (%)	Test (%)
Context size	32 DWT + 16 FFT (48 total)	88.54 ± 0.21	88.68 ± 0.27
Context size	64 DWT + 32 FFT (96 total)	88.79 ± 0.24	88.97 ± 0.18
Context size	96 DWT + 48 FFT (144 total)	89.04 ± 0.17	89.18 ± 0.16
Context size	128 DWT + 64 FFT (192 total)	88.91 ± 0.20	89.07 ± 0.22
Expert count	2	88.60 ± 0.19	88.72 ± 0.26
Expert count	3	89.04 ± 0.17	89.18 ± 0.16
Expert count	4	88.86 ± 0.14	89.01 ± 0.24
Expert count	6	88.61 ± 0.25	88.81 ± 0.20

**Table 11:** Additional design checks on RAF-DB with 30% symmetric noise. Values are mean ± standard deviation over five seeds.

Study	Configuration	Accuracy (%)	Auxiliary Quantity
FFT representation	16-bin radial	89.18 ± 0.16	48-D FFT embedding
FFT representation	Radial + 4 angular sectors	89.23 ± 0.19	52-D FFT embedding
FFT representation	Lightweight 2D spectrum CNN	89.11 ± 0.23	64-D FFT embedding
Path assignment	DWT routing/FFT affine	88.61 ± 0.20	Split context
Path assignment	FFT routing/DWT affine	88.47 ± 0.22	Reversed split
Path assignment	Joint context for both	89.18 ± 0.16	Final design
Classifier	Linear 768–7	89.18 ± 0.16	|val−test| = 0.14%
Classifier	MLP 768–384–7	89.08 ± 0.22	|val−test| = 0.69%
Classifier	MLP 768–384–192–7	88.94 ± 0.27	|val−test| = 0.93%
Branch layout	Unified early multiscale fusion	88.46 ± 0.22	Not parameter-matched
Context access	Raw context only	64.37 ± 0.42	No semantic feature
Context access	Passive context concat	88.02 ± 0.15	No active FEM
Context access	Full FEM + context head	88.75 ± 0.19	Raw context exposed
Context access	Full FEM, no context head	89.18 ± 0.16	Final design

**Table 12:** Inference-time context interventions on RAF-DB. Entries are means over the final validation-selected checkpoint from each of the five principal seed runs; deterministic perturbations are applied within each checkpoint, and this sensitivity table reports mean changes only. “Classifier only” keeps the correct context inside FEM and perturbs only the extra raw-context classifier input.

Model	Perturbed Copy	Correct	Shuffled	Zeroed
Full FEM, no context head	FEM	89.18	87.83	87.69
Full FEM + context head	Classifier only	88.75	87.92	87.78
Full FEM + context head	FEM + classifier	88.75	86.96	86.81

**Table 13:** Protocol-matched mechanism comparison and measured cost. Accuracy uses RAF-DB with 30% symmetric noise. All candidates share the insertion stage, data split, corrupted-label files, seeds, optimization schedule, and model-selection rule; candidate-specific search spaces are reported in Table 3, and measured capacities are not constrained to be identical. Latency reports FP32 batch-1 full-sweep median and P95 under the unified measurement setting. All non-FARM mechanisms are unified Swin-stage reimplementations guided by their public definitions.

Method	Accuracy	Params (M)	GFLOPs	Median ms	P95 ms	Peak MiB
Swin-Tiny baseline	87.58 ± 0.22	27.525	4.5084	14.94	29.14	134.59
Passive DWT + FFT	88.02 ± 0.15	27.538	4.5334	18.61	40.98	138.05
SE reimplementation	87.87 ± 0.21	27.598	4.5085	16.70	43.45	135.21
CBAM reimplementation	88.05 ± 0.23	27.599	4.5086	16.09	44.78	135.21
Frequency-FiLM	88.42 ± 0.17	27.861	4.5337	18.26	62.09	139.31
Dynamic/conditional conv.	88.30 ± 0.24	30.037	4.6244	15.91	47.96	144.51
Visual-feature MoE	88.38 ± 0.26	28.562	4.5095	16.66	57.73	138.89
FARM-FER	89.18 ± 0.16	28.982	4.5349	19.85	60.51	143.56

FLOPs use the same 224 × 224 input setting; latency and memory are measured end to end.

**Table 14:** Illustrative mean routing probabilities and blend gate for 1000 fixed RAF-DB test images under each controlled condition using the seed-31 checkpoint. Standard deviations are across images; the separate five-seed collapse audit is reported in the text.

Condition	Expert 1	Expert 2	Expert 3	Blend β
Sharp/clean	0.47 ± 0.19	0.32 ± 0.17	0.21 ± 0.14	0.61 ± 0.12
Gaussian blur	0.20 ± 0.14	0.54 ± 0.20	0.26 ± 0.16	0.69 ± 0.11
Low contrast	0.24 ± 0.15	0.22 ± 0.14	0.54 ± 0.21	0.42 ± 0.13
JPEG compression	0.31 ± 0.17	0.27 ± 0.15	0.42 ± 0.19	0.48 ± 0.12

**Table 15:** Controlled descriptor sensitivity. Correlations are Spearman ρ with 95% cluster-bootstrap intervals; probe values are held-out $R^{2}$ mean ± standard deviation across five folds.

Factor	DWT Statistic ρ	FFT Statistic ρ	DWT Probe $R^{2}$	FFT Probe $R^{2}$
Local edge density	+0.76 [0.73,0.79]	+0.41 [0.36,0.46]	0.58 ± 0.021	0.29 ± 0.026
Gaussian blur sigma	−0.67 [−0.71,−0.63]	−0.89 [−0.91,−0.87]	0.47 ± 0.024	0.72 ± 0.017
Gaussian-noise sigma	+0.83 [0.80,0.85]	+0.78 [0.75,0.81]	0.65 ± 0.019	0.59 ± 0.023
Contrast reduction	−0.38 [−0.43,−0.33]	−0.71 [−0.75,−0.67]	0.24 ± 0.028	0.51 ± 0.020

**Table 16:** Quantitative representation and ground-truth-class Grad-CAM analysis on RAF-DB. Values are mean ± sample standard deviation across five seed checkpoints. Distances and Fisher ratio are normalized to the backbone value within each seed.

Analysis	Metric	Backbone	Passive Concat	FARM-FER
Representation	Silhouette score	0.181 ± 0.007	0.203 ± 0.005	0.246 ± 0.006
Representation	Normalized intra-class distance	1.000	0.967 ± 0.011	0.918 ± 0.015
Representation	Normalized inter-class distance	1.000	1.026 ± 0.013	1.071 ± 0.014
Representation	Fisher separation ratio	1.000	1.061 ± 0.019	1.167 ± 0.021
Grad-CAM	Insertion AUC (higher)	0.607 ± 0.014	0.619 ± 0.011	0.646 ± 0.012
Grad-CAM	Deletion AUC (lower)	0.409 ± 0.013	0.399 ± 0.015	0.376 ± 0.010
Grad-CAM	Perturbation stability	0.718 ± 0.018	0.735 ± 0.013	0.772 ± 0.015

**Table 17:** Frozen linear probes from pre-modulation v and refined $\tilde{v}$ to controlled frequency condition targets. Values are held-out $R^{2}$ mean ± standard deviation over five folds.

Probe Target	Backbone v	Refined $\tilde{v}$
Gaussian blur severity	0.31 ± 0.021	0.48 ± 0.026
Contrast-reduction severity	0.27 ± 0.029	0.43 ± 0.020
DWT high-frequency energy	0.38 ± 0.024	0.56 ± 0.027
FFT high-frequency ratio	0.35 ± 0.028	0.52 ± 0.022

## Data Availability

This study used three publicly available datasets:RAF-DB [1], FER+ [2], and AffectNet [3]. The access details for each dataset can be found in the corresponding references.
